# RETRACTION: Antinociceptive Activities and the Mechanisms of Anti‐Inflammation of Asiatic Acid in Mice

**DOI:** 10.1155/ecam/9875307

**Published:** 2026-03-03

**Authors:** Evidence-Based Complementary and Alternative Medicine

RETRACTION: S‐S. Huang, C‐S. Chiu, H‐J. Chen, et al., “Antinociceptive Activities and the Mechanisms of Anti‐Inflammation of Asiatic Acid in Mice,” *Evidence-Based Complementary and Alternative Medicine*, vol. 2011 (2011), https://doi.org/10.1155/2011/895857.

The above article, published online on 20 April 2011 in Wiley Online Library (https://wileyonlinelibrary.com), has been retracted by John Wiley & Sons Ltd.

The retraction has been agreed following an investigation of the concerns raised by *Actinopolyspora biskrensis* on PubPeer [1], which identified several concerns related to Figure 8a in the paper. More specifically, panels c and d are identical after a vertical flip, despite representing different tissues. In addition, the image of Carr‐treated group in Figure 8b appears in [2] (after 180 degree rotation) as well as Figure 4a of [3].

As a result of the investigation, the data and conclusions of this article are considered unreliable.

The authors have been informed of this decision but did not provide a response.
